# Functional brain imaging across development

**DOI:** 10.1007/s00787-012-0291-8

**Published:** 2012-06-24

**Authors:** Katya Rubia

**Affiliations:** Department of Child Psychiatry/SGDP, Institute of Psychiatry, P046, King’s College London, De Crepigny Park, London, SE5 8AF UK

**Keywords:** fMRI, Development, Maturation, Cognitive control, Inhibition, Timing, Attention, Motivation, Decision making, Resting state, Default mode network (DMN), Attention, Functional connectivity

## Abstract

The developmental cognitive neuroscience literature has grown exponentially over the last decade. This paper reviews the functional magnetic resonance imaging (fMRI) literature on brain function development of typically late developing functions of cognitive and motivation control, timing and attention as well as of resting state neural networks. Evidence shows that between childhood and adulthood, concomitant with cognitive maturation, there is progressively increased functional activation in task-relevant lateral and medial frontal, striatal and parieto-temporal brain regions that mediate these higher level control functions. This is accompanied by progressively stronger functional inter-regional connectivity within task-relevant fronto-striatal and fronto-parieto-temporal networks. Negative age associations are observed in earlier developing posterior and limbic regions, suggesting a shift with age from the recruitment of “bottom-up” processing regions towards “top-down” fronto-cortical and fronto-subcortical connections, leading to a more mature, supervised cognition. The resting state fMRI literature further complements this evidence by showing progressively stronger deactivation with age in anti-correlated task-negative resting state networks, which is associated with better task performance. Furthermore, connectivity analyses during the resting state show that with development increasingly stronger long-range connections are being formed, for example, between fronto-parietal and fronto-cerebellar connections, in both task-positive networks and in task-negative default mode networks, together with progressively lesser short-range connections, suggesting progressive functional integration and segregation with age. Overall, evidence suggests that throughout development between childhood and adulthood, there is progressive refinement and integration of both task-positive fronto-cortical and fronto-subcortical activation and task-negative deactivation, leading to a more mature and controlled cognition.

## Introduction

Complex cognitive functions that are important for mature goal-directed adult behaviour develop throughout adolescence and well into adulthood [1–3]. These so-called “executive functions” (EF) include motor and interference inhibition, performance monitoring, cognitive flexibility, attention control, planning, decision-making, temporal foresight and working memory [4]. Cognitive control refers more specifically to functions that require the overriding of interfering responses, such as motor and interference inhibition, performance monitoring and executive attention control [5]. A recent distinction has been made between “cool” and “hot” EF, whereby “cool” cognitive executive functions (EF) refer to relatively abstract and dyscontextualized functions, such as the functions traditionally labelled as EF or cognitive control, as defined above, while “hot” EF involve monetary reward or incentives, such as reward-related decision-making and gambling tasks [6]. Top-down control of motivation is an essential indicator of adult mature behaviour and develops still between adolescence and adulthood with adolescents being more risk-friendly and having less control over the impact of proximal rewards than adults [7–9]. There are other functions, however, which also develop relatively late and are less well researched. For example, timing functions, including motor timing, time estimation and temporal discounting are further refined throughout late childhood and adolescence [9–12]. Many of these timing functions are essential to executive functions such as temporal foresight (crucial for planning and decision-making), time estimation and motor timing (underlying mature adult time management) and fine-temporal discrimination (important for speech/phoneme perception) [13, 14]. However, even simpler cognitive functions such as selective and visual–spatial attention functions are thought to be progressively refined during late childhood and adolescence [15–17].

Adolescence is typically defined as the developmental stage occurring between puberty and legal adulthood and varies between individuals. Most developmental imaging studies, however, have used a cut-off of age 13 and above to define adolescence as opposed to childhood that is usually considered to reach ages up to 12 years. Sex differences become more pronounced during this period of adolescence, concomitant with the hormonal changes of puberty [18] with females being more efficient than males in tasks of selective attention, verbal fluency and conductive reasoning [2, 16], and males outperforming females in cognitive functions that rely on visual–spatial processing, especially mental rotation [1, 19].

During this time, and presumably underlying these changes, the brain continues to mature via processes such as synaptic remodelling and competitive elimination, programmed cell death and myelination [20, 21]. Structural imaging studies demonstrate a linear increase with age in white matter, presumably reflecting myelination, that peaks at around age 50, and a non-linear decrease in grey matter density and cortical thickness, presumably reflecting synaptic pruning and myelination, up to age 40 [22, 23]. These processes are heterochronous and heterogeneous with higher association areas in frontal, parietal and temporal regions maturing latest and primary sensory areas maturing earliest [22, 24]. Gender differences show that males exhibit steeper developmental slopes in grey matter reduction and white matter increase than females, partly explained by earlier maturation peaks in females in frontal, striatal, temporal and parietal areas [25, 26] Gender differences in cognitive abilities may at least in part be explained by these sex differences in brain structure and its development [25].

In line with structural development, functional magnetic resonance imaging (fMRI) studies show progressive linear increases with age between childhood and adulthood in the activation and functional inter-regional connectivity of task-relevant fronto-striatal, fronto-temporo-parietal and fronto-limbic networks mediating these late developing cognitive functions, well into the third and fourth decade of life [27–35]. Emerging evidence also points at gender differences in the age-associated changes [28, 30, 35]. The fMRI literature on resting state connectivity shows that this progressive age-associated increase in activation and inter-regional connectivity is associated with progressively stronger deactivation of the task-anti-correlated resting state network, both of which are associated with more mature performance [30]. Furthermore, children have more short-range connections which are progressively replaced by longer range connections in adulthood, suggesting that development is characterised by both progressive integration and segregation [36, 37]. This review summarises the current evidence for age-associated changes in brain function between childhood and adulthood during these late developing functions of cognitive and motivation control, attention and timing functions as well as of resting state networks.

## Functional maturation of cognitive control

**Fig. 1:**
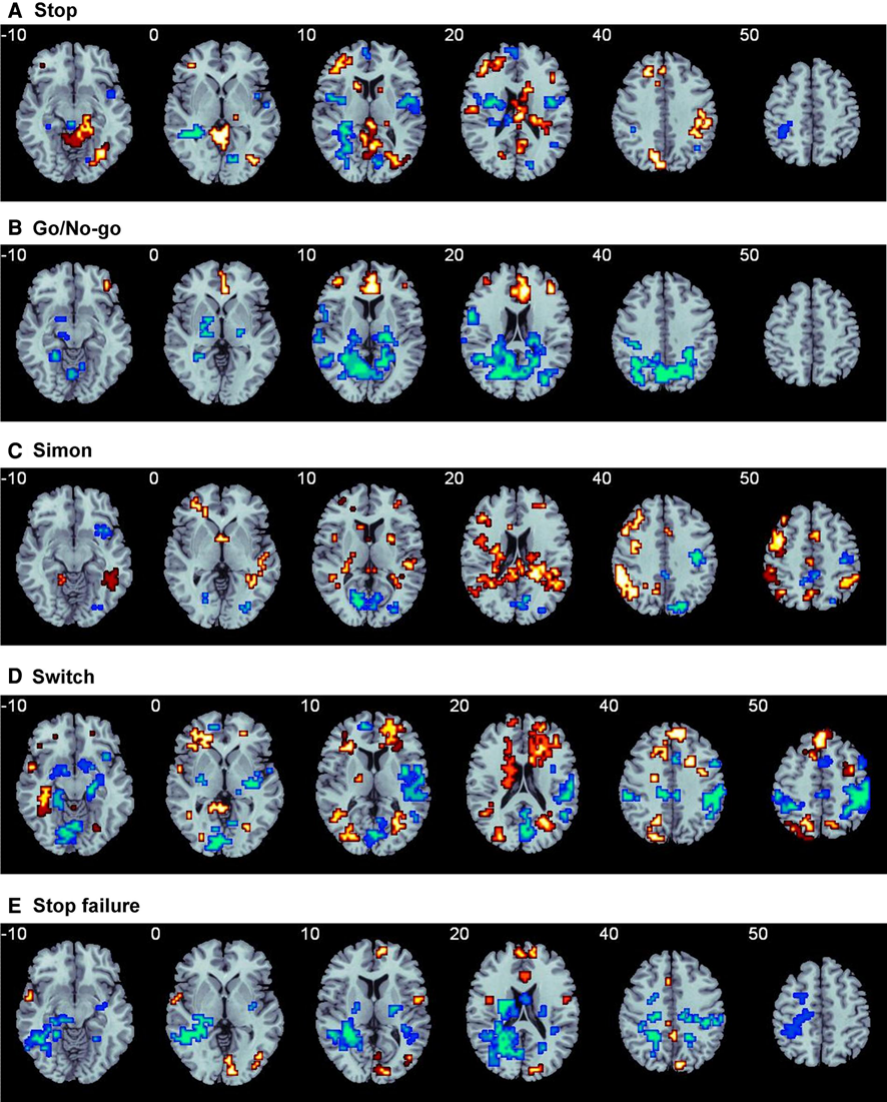
The figure shows areas that progressively increase in activation with age (*orange*) and areas that progressively decrease (*blue*) in activation with age between late childhood and adulthood (10–43 years) in a range of cognitive control tasks. Task-relevant frontal and/or fronto-striatal regions increase progressively with age in their activation while posterior and limbic areas showing negative age correlations. **a** Stop motor response inhibition task: left dorsal and inferior lateral as well as medial prefrontal areas, striato-thalamic and parietal activation increases linearly with age [27]. **b** Go/No-Go motor response inhibition task: medial and lateral frontal activation increases linearly with age [42]. **c** Simon (interference inhibition) task: predominantly left dorsolateral and inferior prefrontal as well as medial prefrontal activation together with striato-thalamic and temporo-parietal activation increases progressively with age [28]. **d** Switch task (cognitive flexibility): Bilateral dorsolateral and inferior prefrontal, medial frontal and striato-thalamic activation increases progressively with age [28]. **e** Performance monitoring (Stop failures): anterior cingulate and medial frontal together with superior temporal activation increases linearly with age [27] (color figure online)

Functions of cognitive control, including motor and interference inhibition, cognitive switching and performance monitoring, develop late in childhood and adolescence [1, 3, 38] and are mediated by lateral inferior prefrontal cortex (IFC), anterior cingulated cortex/supplementary motor area (ACC/SMA), basal ganglia, and parieto-temporal regions [39, 40]. There is consistent evidence for progressive age-correlated increase of activation in task-relevant lateral and medial fronto-striatal brain regions and in the strength of their inter-regional connectivity during tasks of cognitive control. During withdrawal of an already planned response in the Stop task, progressive increase of activation as well as of inter-regional connectivity, as shown by time-course correlation analyses, was observed in the age range of 10–42 years in a typical inhibition network of inferior fronto-striato-thalamic and cerebellar regions, which was furthermore correlated with faster motor inhibition speed [27] (Fig. 1a). Findings survived when performance-matched subgroups were compared, suggesting that changes were truly age and not just performance-related. Similar findings of progressively enhanced activation with age in lateral and medial frontal regions were observed during Go/No-Go task performance between the age range of 7–22 years [41] and 10–43 years [42]. The first study did not control for performance differences. However, in the study of Rubia et al., [42], findings survived covariance for performance, suggesting true age-related changes. In line with the age-regression findings, a categorical comparison showed enhanced activation in lateral and medial frontal as well as parietal regions in adults between 19 and 33 years relative to children between 8 and 12 years during the Go/No-Go task, which survived in the comparison of performance-matched subgroups [43]. Furthermore, there was evidence for progressively negative age-correlated activation in earlier developing posterior occipital and infero-temporal areas between 10 and 43 years [42] (Fig. 1b). During tasks of interference inhibition, prominently inferior but also DLPFC-striatal, anterior cingulate and parieto-temporal regions that are known to mediate interference inhibition [44] were progressively more recruited with increasing age, which was associated with better task performance, while medial frontal and posterior areas were progressively negatively age correlated [28, 30, 42, 43, 45, 46] (Fig. 1c). Furthermore, the age-associated findings were not confounded by performance differences, given that groups were either naturally matched in performance [45] or findings survived performance-matched subgroup analyses or performance-covariation [28, 30, 42, 43, 46]. While the majority of studies found linear effects to account best for the developmental changes, one of these studies, testing adolescents and adults between 14 and 25 years found that curvilinear functions accounted better for the activation changes than linear functions; frontal activation increases peaked at 21 years and then declined again [45]. A purely paediatric study found increased activation in adolescents (14–15 years) relative to children (8–11 years) in left inferior parietal lobe after covarying for performance differences [47]. During cognitive switching a similar picture emerges where bilateral inferior fronto-cingulo-striato-parietal areas that are typical for adult switching [40] were progressively more recruited between late childhood and adulthood between 10 and 43 years which correlated with a more reflective performance [28, 42] (Fig. 1d). Findings persisted when performance was covaried, suggesting true age effects [28, 42]. Four studies used categorical age comparisons between relatively small groups of children and adolescents and adults. Two studies showed enhanced activation during switching tasks in adults between 18 and 32 years in parieto-temporal and thalamic regions relative to children of 7–11 years [48] or children and adolescents between 11 and 13 years [49]. However, one study found that adults had enhanced right superior frontal activation while the other study found that children had enhanced activation in this region [49]. The third study showed higher switch costs in children and more immature activation patterns in pre-SMA and VLPFC in children aged 8–12 years and in VLPFC in adolescents aged 13–17 years relative to adults between 18 and 25 years [50]. Similarly, enhanced inferior and medial frontal as well as parietal and cerebellar activation was observed for adults between 22 and 40 relative to children and adolescents between 10 and 16 during an inhibition change task [51]. Performance was only covaried in two of these studies [49, 51].

Laterality findings were also observed, with progressive left-lateralisation in older subjects or enhanced left frontal activation in adults relative to children [28, 43, 52]. However, as mentioned above, one study observed enhanced right superior frontal activation in children between 11 and 13 years relative to adults in a relatively small sample [49]. One of the first studies to investigate developmental changes in functional inter-regional connectivity networks during inhibition using independent component analyses, found that adolescents between 11 and 17 years relative to adults of 18–35 years had decreased connectivity in several inhibitory networks, including a medial network comprising anterior cingulate, inferior frontal lobe and parietal regions, as well as a more lateral IFC-striato-thalamic network, although some regions were strongly recruited in adolescents such as ventrolateral prefrontal lobe [33]. Furthermore, coupling between segregated inhibitory networks was reduced in adolescents [33], suggesting progressive functional integration within and between executive circuitries. During interference inhibition, using a psychophysiological interaction analysis, increased functional connectivity was observed within fronto-thalamic and fronto-parietal networks in adolescents between 14 and 15 years relative to children between 8 and 11 years [47]. An interesting meta-analysis of cognitive control studies, including studies of children between 6 and 13 years and studies that were defined as adolescent studies, including children and adolescents between 10 and 17 years, found consistent activation in IFC/DLPFC and anterior insula in all groups with a laterality age effect, however, in the anterior insula which was more activated in the right hemisphere in adolescents relative to children [53], presumably reflecting increased cognitive control. Similar findings were also observed for error monitoring in two independent studies. Increasing age between 10 and 42 years was correlated with progressively enhanced activation in a typical performance monitoring network [54] of anterior and posterior cingulate during response inhibition errors [27] (Fig. 1e). Findings remained when performance was covaried. Similar findings of increased activation in adults relative to children aged 8–12 years and adolescents aged 13–17 years in ACC during errors of reflex inhibition were also observed by a second study, in which performance did not differ across age groups [55]. A study using functional inter-regional connectivity using independent component analysis during performance monitoring showed that similar error processing networks are active in adolescents and adults between 11 and 37 years, with, however, progressively enhanced activation in adults in several regions of these networks, including anterior cingulate, lateral frontal, striato-thalamic and parietal areas [34]. Together, the three studies show that anterior cingulate-striato-thalamic performance monitoring networks still mature between childhood and adulthood, just like lateral prefronto-striato-parietal cognitive control networks.

Overall, the findings suggest that cognitive control networks that mediate motor and interference inhibition and cognitive flexibility, comprising IFC, the basal ganglia and their connections to temporo-parietal regions [56] as well as medial PFC-striato-thalamic networks of performance monitoring are progressively more recruited with increasing age between childhood and adulthood. This progressive activation increase in task-relevant networks, furthermore, is associated with more mature performance and is yet truly age associated, as shown by those studies that controlled for developmental performance differences.

## Functional maturation of timing functions

The functional maturation of timing functions is relatively understudied, despite consistent evidence for relatively late maturation of these functions [9, 11, 57] and the fact that they are essential for other late developing executive functions such as planning (temporal foresight), speech (time discrimination) and mature time management (motor timing and time estimation) [13, 14]. Timing functions are typically subcategorized into (1) motor timing, measured in tasks that require the adjustment of a motor response to externally given temporal intervals, typically of milliseconds or seconds; (2) time perception, measured in tasks requiring to estimate, (re)produce or discriminate between temporal intervals between milliseconds to minutes; and (3) temporal foresight, testing forward thinking and the future consideration of current behaviour, measured in temporal discounting or inter-temporal choice tasks of typically longer interval ranges of weeks to years. They require inter-temporal bridging of relatively long temporal distances and are important for decision-making, future planning or inter-temporal choice behaviour.

**Fig. 2:**
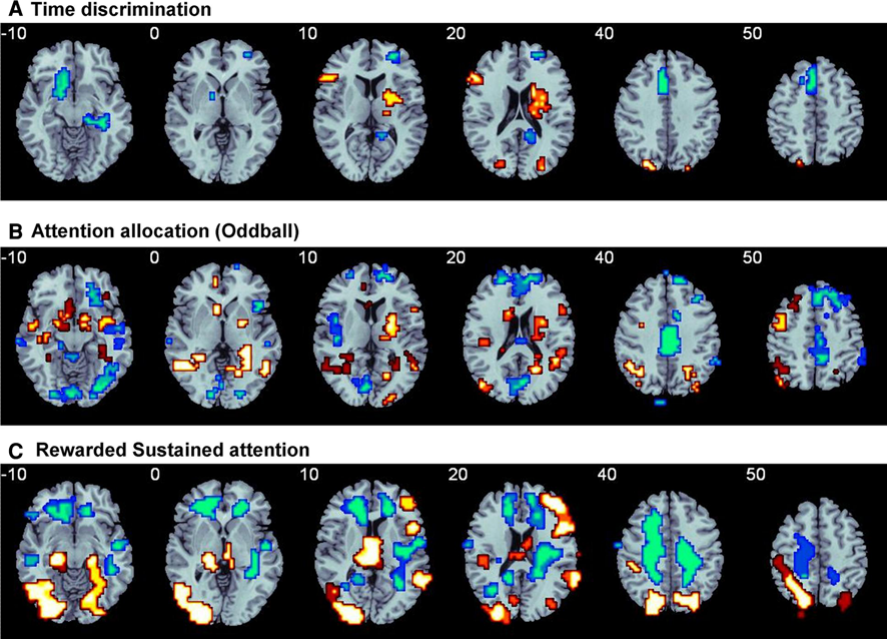
The figure illustrates areas that progressively increase in activation with age (*orange*) and areas that progressively decrease (*blue*) in activation with age between late childhood and adulthood (10–43 years) during tasks of attention and timing. A shift from medial to lateral frontal activation is observed during the two attention and time estimation tasks. **a** Time discrimination: progressively increased recruitment with age of left lateral prefrontal activation with diminishing recruitment of medial frontal activation [31]. **b** Attention allocation in an Oddball task: progressively more right and left lateral frontal and striatal areas are recruited with increasing age, with diminishing recruitment of medial frontal activation [35]. **c** Rewarded sustained attention task in a continuous performance task: progressively more right dorsal and inferior lateral prefrontal regions are recruited with increasing age with diminishing recruitment with age in medial frontal activation [32]. The reduction in medial frontal regions may also reflect enhanced deactivation of the default mode network (color figure online)

The first developmental fMRI study of motor timing processes found that during sensorimotor synchronisation of 6 s, 8 adults performed better than 9 children and adolescents between 10 and 17 years and showed increased activation in anterior and posterior cingulate, right IFC, putamen and left inferior parietal lobe. Furthermore, there was a significant linear activation increase with age in right IFC as well as in the anterior and posterior cingulate activations [52]. Performance, however, was not correlated with the activation changes. Two fMRI studies investigated the functional maturation of fine-temporal perception, known to be mediated by lateral frontal, striatal and parietal brain regions [13, 14, 58]. In a narrow age range of children and adolescents between 10 and 15 years no developmental change in activation or in functional inter-regional connectivity was observed for time discrimination in the millisecond range [47]. A larger study on 32 participants between 10 and 53 years found that despite no significant age effects on time discrimination task performance (although children were slightly more inaccurate), there were significant linear age correlations in typical regions of time estimation such as left DLPFC and IFC, insula, striatal and superior parietal areas [31] (Fig. 2a). Striatal activation in particular correlated positively with both age and time discrimination performance, in line with a pivotal role for the striatum in temporal perception [58, 59]. Furthermore, adults compared to children and adolescents showed significantly enhanced functional connectivity, as tested using correlational connectivity analyses of the time-courses of activation of seed regions, between left and right fronto-striatal activation and between right frontal and parietal activations. Progressively decreased activation with age was observed within midline paralimbic areas such as ventromedial prefrontal cortex (vmPFC), anterior and posterior cingulate and cerebellum, suggesting age-dependent developmentally dissociated neural networks for time discrimination, with children relying more on earlier developing limbic and cerebellar regions, and adults shifting towards increased activation and better functional inter-regional connectivity in more specialised left-hemispheric lateral fronto-striatal and fronto-parietal activation [31].

Only one study tested for functional maturation of temporal foresight in a temporal discounting task where participants have to choose between an immediate but smaller reward and a larger but delayed reward. The task measures the degree to which a reward is being subjectively discounted in proportion to the delay, thus quantifying the individual sensitivity to the timing/delay of a reward and the capacity of temporal foresight to understand the future gain of the delayed choice. They hence require inter-temporal decision-making, future reward evaluation and inter-temporal bridging. Because the task also measures reward processing and reward-related inter-temporal decision-making (even if the key dependent variable is the timing of the reward), it is often categorised a “hot” executive function. Steeper temporal discounting in the task has been associated with impulsiveness and is thought to reflect ‘myopia for the future’, i.e., problems with temporal foresight [29, 60].

The developmental imaging study tested 40 subjects between 10 and 43 years in a temporal discounting task [29]. With increasing age there was progressively decreasing steepness of temporal discounting of the subjective value of the reward with increasing delays. Lateral fronto-striato-cerebellar and parieto-temporal regions were significantly positively age-correlated, while medial limbic cortico-striatal areas were negatively age-correlated [29]. Importantly, there was an interaction between reduced discounting and age, where both enhanced age and reduced discounting was associated with increased activation in ventromedial orbitofrontal cortex (vmOFC) and progressively decreased activation in ventral striatum, a region that processes proximal rewards [61]. Increasing age was furthermore associated with progressively enhanced inter-regional connectivity between vmOFC and ventral striatum suggesting progressive top-down inhibition of the more immature limbic hyper-responsiveness to immediate rewards, leading to a more mature decreased discounting style. In addition, there was progressively increased functional inter-regional connectivity with age between performance and age-correlated vmOFC and lateral inferior fronto-insular and parietal connections during delayed choices, suggesting progressively stronger recruitment and activation coherence of vmOFC with lateral fronto-parietal temporal foresight mechanisms to support longer-term foresighted decisions [29]. The findings are in line with the notion that limbic hyper-responsiveness to reward in children is progressively more tempered by late maturing prefrontal control systems [29].

In conclusion, it appears that during tasks of temporal processes, including motor timing, time estimation and temporal foresight, task-relevant frontal brain regions, such as anterior cingulate and inferior frontal cortex for motor timing, ventromedial and lateral prefrontal regions for temporal foresight and lateral inferior prefrontal areas for time estimation are progressively more recruited between childhood and adulthood and show progressively strengthened inter-regional connectivity with underlying striatal and parietal connections, leading to more mature cognitive time management.

## Functional maturation of attention functions

Relatively few fMRI studies have investigated attention control functions, despite the fact that attention control is also progressively refined throughout late childhood and adolescence [1, 17, 62]. Attention control refers to the individuals’ capacity to selectively attend some stimuli while ignoring others [63] which hence comprises selective attention and sustained attention, defined as the ability to voluntarily maintain the focus of attention to infrequently occurring critical events [64]. A developmental fMRI study of a relatively simple function of selective attention to rare oddball trials among a string of frequent standard trials showed significant age-associated activation increases in lateral fronto-striatal and temporo-parietal regions across 63 participants between 13 and 38 years, while decreasing age was associated with more activation in midline frontal, mid-cingulate and occipito-cerebellar regions [35] (Fig. 2b). Progressive age was furthermore associated with a speed accuracy trade-off, favouring accuracy, sacrificing speed, suggesting a less impulsive performance style in adults. Performance in this study, however, was not covaried. The findings show that functional activation increases in fronto-striatal and temporo-parietal areas are also underlying relatively simple functions such as perceptive attention allocation [35]. A comparison between children between 8 and 12 years and adults during alerting and reorienting showed that children showed reduced activation in a priori defined regions of interest, i.e., in right fronto-cingulate and midbrain regions of alerting together with smaller alerting effects, and reduced activation in the right temporo-parietal junction during reorienting, concomitant with higher reorienting costs. Furthermore, findings remained in performance-matched subgroup analyses [46]. A developmental fMRI study of sustained attention in the continuous performance task in a large sample of 70 children and adults between 10 and 43 years showed progressively stronger recruitment in older subjects of brain regions associated with sustained attention in right-hemispheric lateral inferior frontal, superior temporal and inferior parietal cortices, despite comparable task performance [32]. Furthermore, linear age-associated activation decreases were observed in earlier developing limbic and paralimbic medial temporal, posterior insular and posterior cingulate regions, suggesting stronger recruitment of bottom-up saliency detection processes in children and stronger recruitment of top-down attention control processes in adults [32]. Interestingly, when monetary reward was added to the attention trials, reward further potentiated the age-dependent activation increases in the sustained attention network in inferior frontal, temporal and cerebellar brain regions and elicited additional activation increases within top-down executive attention and motivation control areas such as dorsolateral (DLPFC) and vmPFC and dorsal striatum, presumably leading to more effective integration of motivation and cognition (Fig. 2c). With decreasing age, however, reward also amplified the earlier developing, more primitive posterior bottom-up visual spatial saliency detection regions that were negatively age-correlated [32]. The findings show that incentives have age-dependent effects on the development of attention networks, increasing activation in developing attention control and executive reward-processing regions, whilst decreasing paralimbic networks of visual–spatial attention to motivational saliency.

## Functional maturation of motivation control

“Hot” executive functions, such as reward-related decision-making tasks, measure the impact of incentives or motivation on cognitive performance. Motivation in this context refers to internal (i.e., ambition) or external (i.e., incentives) factors that stimulate interest in good cognitive performance [61]. “Hot” EF are mediated by vmOFC/vmPFC, anterior cingulate and limbic brain structures, where prefrontal regions appear to be closely interconnected with and exert top-down control over limbic areas of reward response, leading to mature decision-making [61]. There is evidence for enhanced risk-taking in adolescence, which may be associated with the late development of top-down executive control mechanisms over more immature reward hypersensitivity [9]. Developmental functional imaging studies of reward-related decision-making tasks observed enhanced OFC activation in children aged 9–11 years and adolescents aged 13–17 years relative to adults aged 23–29 years [65] despite no marked group differences in performance, and enhanced ventral striatal activation in adolescents relative to adults and children during reward outcome [65, 66]. During comparable performance of a monetary, two-choice decision-making task with varying levels of risk and rewards, enhanced activation was shown in adults aged 20–40 relative to adolescents aged 9–17 years in a priori selected regions of interest in dorsal ACC and OFC, which, furthermore correlated with less risk-friendly performance, while children activated more ventral ACC [67]. One study, however, found greater activation of the dorsal ACC and no differences in vmOFC and DLPFC activation in children (9–12 years) compared to adults (18–26 years) for high-risk relative to low-risk choices and greater recruitment of lateral OFC in children for negative feedback in a relatively simple choice selection task based on outcome probability [68]. Given that performance was only marginally different, the enhanced dorsal ACC activation in younger subjects was interpreted as greater effort for conflict inhibition in children and the enhanced lateral OFC activation as increased sensitivity to negative feedback.

While developmental activation changes in OFC and ACC have been inconsistent during reward-related tasks, studies seem to converge, however, in that ventral striatum is more activated in adolescents than adults during reward outcome, suggesting heightened sensitivity to rewards in adolescence that could potentially explain higher risk-taking behaviours [65, 66, 68]. Recent studies highlight non-linear developmental changes in the activation of motivation control regions. Thus, during a decision-making task, a peak was observed in adolescence (12–17 years) relative to childhood (8–10 years) and adulthood (19–26 years) for vmPFC activation during the choice phase and a peak in VS during the outcome phase of the task, despite comparable task performance [69]. In line with this are findings that during happy No-Go trials, known to elicit approach behaviour, adolescents (13–17 years), relative to both children (6–12 years) and adults (18–29 years) made more commission errors and showed heightened activation in ventral striatum that was not observed during neutral No-Go trials and that survived correction for performance differences [70]. Overall, the findings suggest that development of motivation control is associated with non-linear changes, where adolescents show exaggerated hyper-response in reward-processing regions which may not yet be tempered by prefrontal top-down regulatory structures leading to more risk-friendly decision-making behaviour [9].

## Sex by age interactions

Very few developmental imaging studies had the power to test for age by sex interactions. Two fMRI studies show that gender differences in activation may be the result of underlying differences in the functional development of these sex-dimorphic regions. A developmental imaging study of attention allocation in an oddball task across the age range of 13–38 years, showed that females had sex-specific age correlations in right fronto-striato-temporal regions—that were enhanced in activation in all females relative to all males—while males had male-specific age correlations in left medial temporo-parietal areas that were increased in activation in all males relative to all females, independently of age [35]. Strikingly similar findings of female-exclusive age correlations in inferior and medial prefrontal regions and of male-exclusive age correlations in superior parietal and temporal regions were observed for the same group of subjects in the age range of 13–38 years during interference inhibition and switching [28]. Similarly, the sex-specific age-correlated activations overlapped with sex-dimorphic activation patterns. Together, the findings suggest that sex differences in the recruitment of frontal (increased in females) and parietal areas (increased in males) during tasks of cognitive control and attention are associated with underlying differences in the functional maturation of these brain regions. Similarly, during another interference inhibition task, conducted across a large age range between 7 and 57 years, female-specific age correlation effects were observed for the caudate [30]. In addition, the study also observed sex by age interaction effects in the anterior cingulate, which is part of the default network, with males showing progressively more deactivation with age in this region [30].

In summary, it seems that sex differences in the form or enhanced recruitment of fronto-striatal regions in females and enhanced parieto-temporal activation in males during tasks of cognitive control [28, 35], may be based on underlying differences in the functional maturation of these regions.

Given that across all subjects, inferior frontal and striatal regions were positively age-correlated, females appeared to show the more mature activation pattern in these regions, while in parietal areas males showed the more mature activation patterns, since these areas were age-correlated in all subjects [28, 30, 35].

The finding of more mature frontal activation in females is in line with structural evidence of earlier morphometric development of these regions in females [25, 71, 72]. Similarly, the parietal lobe has been shown to be larger in grey matter volume in males [73].

These developmental age by sex interaction findings thus demonstrate that sex differences in task-related brain activation appear to be related to sex differences in the underlying progressive functional development of these brain regions, paralleling structural sex-dimorphic developmental differences, and stressing the importance of including developmental data when investigating gender effects on neural brain activation.

## Developmental functional connectivity studies

As mentioned, during cognitive tasks there is consistent evidence for enhanced inter-regional connectivity in adults compared to children in task-relevant fronto-frontal, fronto-striatal, fronto-parietal and fronto-cerebellar networks of cognitive control, timing and motivation functions [27, 29, 31–34].

Recent interest has been on the resting state or default mode network (DMN). The DMN consists of intercorrelated co-activation of medial frontal lobe, anterior and posterior cingulate and inferior temporal and parietal areas during rest, that are parametrically attenuated during cognitive load, presumably reflecting increases in attentional and computational resources that impinge upon task-unrelated thoughts and processes. The DMN is therefore thought to reflect self-referential and stimulus-independent thought processes that need to be switched off for successful cognitive functioning, which is shown by increased attentional lapses being associated with a failure to inhibit the DMN [74].

Developmental neuroimaging studies of the DMN show that children as young as 1–2 year old show a resemblance of the DMN [75, 76]. Older children between 7 and 12 also show rest-related or task-related DMN activity that is parametrically attenuated with increasing task demands. During the resting state, however, not only DMN networks can been observed, but also resting state intrinsic networks of cognitive control, visual, auditory or sensorimotor functions [77]. Using seed-based functional connectivity and graph theoretical metrics, children (7–9 years) when compared to adults have significantly weaker functional connectivity in the anterior and posterior parts of the DMN (mPFC and PCC) as well as in several resting state intrinsic cognitive networks, while adolescents (10–15 years) were intermediate between children and adults [36, 37, 78, 79]. Linear changes are also observed with progressive deactivation in the DMN between 7 and 57 years [30]. The progressive age-associated deactivation of medial frontal DMN regions could also potentially account for the shift of medial to lateral frontal activation observed in some developmental studies. Functional connectivity studies furthermore show that children (8–12 years) have more short-distance connections while adults develop more long-distance connections, for example, between DLPFC and parietal regions or between frontal and cerebellar regions for cognitive control networks or between medial frontal and posterior cingulate and parietal regions for the DMN, with adolescents (13–17 years) showing intermediate patterns [36, 37, 80, 81]. This suggests progressive functional development of the DMN as well as of cognitive control networks between childhood and adulthood through simultaneous progressive integration (increased distributed long-distance connections) and segregation (diminished local short-distance connections), both of which are likely related to progressive myelination and synaptic pruning, respectively [82, 83]. Progressive strength of the functional inter-regional connectivity of both intrinsic cognitive control networks and task-negative DMN is likely associated with progressive cognitive maturation [84]. An impactful recent study using multivariate pattern recognition analysis on brain development using resting state fMRI data on 238 subjects between 7 and 30 years showed that maturation was predicted by a non-linear pattern of increasingly longer range connections (integration) along the anterior posterior axis and progressively decreasing short-range connections (segregation) which peaked at age 22, whereby segregation was a better maturation predictor than integration [85]. Within the classification networks, right anterior prefrontal cortex and precuneus connections were the best predictors for brain maturity [85]. Interestingly, while there are significant correlations between structural and functional connectivity, functional connectivity is not entirely accounted for by structural connectivity since functional interconnectivity can be indirect [86]. The progressive increase in functional connectivity during the resting state as well as during cognitive maturation suggests that “progressive integration through progressive synchronization” [86] underlies the development of both closely interconnected task-positive and task-negative brain networks to provide mature cognitive performance.

## Conclusions and future directions

In conclusion, there is substantial evidence in the developmental cognitive neuroscience literature that cognitive maturation is associated with progressive increases in the activation of task-relevant prefrontal brain regions and their connections to striatal and parieto-temporal regions that mediate top-down control in the context of inhibitory, attention, motivation control and timing functions (Fig. 1). Within frontal regions, there is also some evidence for a shift from earlier developing vmPFC/ACC areas to a more lateralised frontal specialisation in later developing DLPFC/IFC for some functions such as cognitive control, timing and attention [29–32] (e.g., Fig. 2a–c) and some evidence for developmental left-lateralisation effects within frontal regions for some cognitive control and timing functions [28, 29, 31, 52] (e.g., Figs. 1a, c, 2a). Although the majority of studies show increased frontal activation with age, the literature is not entirely consistent, with some studies showing increased activation in children relative to adults in some frontal regions, which has been associated with increased effort for task performance [55, 87]. Functional connectivity studies [27, 29, 31, 33, 34] show that not only the activation but also the functional connectivity of task-relevant brain networks increases with age. Resting state fMRI studies furthermore show that this progressive increase in activation and functional connectivity in task-relevant brain networks is accompanied by progressively stronger deactivation of the task-anti-correlated default mode network [30]. Furthermore, there is consistent evidence that brain development is associated with progressively stronger integration in the form of long-range connections between regions with diminishing short-range connections (i.e., more segregation) suggesting a shift from “local to distributed” organisation [36, 37, 80, 81, 85].

While fMRI studies during tasks of cognitive control have largely shown that progressive increase in activation and inter-regional connectivity within task-relevant fronto-cortical and fronto-subcortical networks is associated with cognitive maturation, the inter-relationship between resting state connectivity development and cognitive maturation needs future investigation [37].

Linear changes appear to be the best models of developmental change in several studies between late childhood and adulthood [27–32, 35, 42, 52]. However, there is also substantial evidence for non-linear changes in studies including lower age ranges. For example, inverted U-shaped activations between childhood and adulthood with peaks in adolescence have been observed [33, 34, 70, 74] which need to be further investigated in future studies of larger sample sizes. The majority of functional developmental imaging studies have controlled for performance differences in order to establish true age effects. However, very few studies have controlled for other factors that are likely to influence brain development, such as differences in pubertal status or sex hormones. Also, so far, only three studies have examined age by sex effects, which are fundamental to understand the developmental underpinnings of sex-dimorphic differences. Lastly, functional MRI studies have been largely cross-sectional. Longitudinal imaging studies are necessary to avoid confounds of cohort effects inherent to cross-sectional studies.

These relatively late functional maturation changes between childhood and adulthood in fronto-striatal and fronto-parieto-temporal networks that mediate these “cool” and “hot” EF are parallel to structural developmental changes over this time period in these regions. In particular, frontal and temporo-parietal association areas as well as the basal ganglia develop relatively late throughout adolescence, decreasing in grey matter and cortical thickness, while increasing in white matter, well into mid-adulthood [22, 23, 88]. Nevertheless, few imaging studies have investigated the common development of structural and functional maturation within the same subjects. Progressively enhanced white matter integrity over development has been associated with better task performance during cognitive control tasks [21, 89] and with activation in adjacent grey matter regions [89]. A study on language abilities, reported that cortical thinning in fronto-parietal regions, associated with cognitive maturation [22, 26] was correlated with stronger fronto-parietal activation [90]. Also, the development of long-distance white matter connections has been shown to play an important role in the development of functional neural networks for executive control, both of which were associated with better performance [34]. There is thus evidence that protracted structural development is associated with more mature brain activation patterns, both of which appear to predict cognitive maturation. Future larger-scale multimodal and longitudinal neuroimaging studies will be necessary to further shed light on the association between structural and functional brain development, avoiding the confounds of cohort effects inherent to cross-sectional studies.

Understanding normal brain development has crucial implications for the understanding of its deviance in neuro-developmental disorders. For example, in autism, studies have shown enhanced short-range connections and reduced long-range connections [91]. In ADHD, abnormal function and functional inter-regional connectivity has been observed in the same fronto-striatal and fronto-cortical networks [92, 93] that are reviewed here to develop progressively with age in their activation and inter-regional functional connectivity, which would be in line with a delay in structural brain maturation [94]. More recently applied multivariate pattern recognition analyses to fMRI may be more sensitive than traditional univariate analyses methods to study the evolution of distributed activation patterns across development and could be used to predict maturation in individual subjects [85]. These methods could potentially distinguish children at different developmental stages of cognitive maturation, which may be relevant to establish whether psychopathologies suffer from a maturational delay, like attention deficit hyperactivity disorder (ADHD) [95] or from a deviance from normal development.
